# Aerosol inhalation of a hydrogen-rich solution restored septic renal function

**DOI:** 10.18632/aging.102542

**Published:** 2019-12-16

**Authors:** Weifeng Yao, Anshun Guo, Xue Han, Shan Wu, Chaojin Chen, Chenfang Luo, Haobo Li, Shangrong Li, Ziqing Hei

**Affiliations:** 1 Department of Anesthesiology, The Third Affiliated Hospital of Sun Yat-sen University, Guangzhou 510630, China; 2 Department of Anesthesiology, The Fifth Affiliated Hospital of Sun Yat-sen University, Zhuhai 519000, China; 3 Department of Anesthesiology, Sun Yat-sen Memorial Hospital, Sun Yat-sen University, Guangzhou 510000, China; 4 Department of Anesthesiology, Affiliated Hospital of Guangdong Medical University, Zhanjiang 524023, China

**Keywords:** hydrogen-rich solution, acute kidney injury, inhalation, sepsis, macrophage

## Abstract

Sepsis-related acute kidney injury (AKI) is known to be caused by inflammation. We explored the renal protective effects of aerosol inhalation of a hydrogen-rich solution (HRS; hydrogen gas dissolved to saturation in saline) in a mouse model of septic AKI. Septic AKI was induced through 18 hours of cecal ligation and puncture. AKI occurred during the early stage of sepsis, as evidenced by increased blood urea nitrogen and serum creatinine levels, pathological changes, renal fibrosis and renal tubular epithelial cell apoptosis, accompanied by macrophage infiltration and M1 macrophage-associated pro-inflammatory cytokine (*Il-6* and *Tnf-α*) generation in renal tissues. Aerosol inhalation of the HRS increased anti-inflammatory cytokine (*Il-4* and *Il-13*) mRNA levels in renal tissues and promoted macrophage polarization to the M2 type, which generated additional anti-inflammatory cytokines (*Il-10* and *Tgf-β*). Ultimately, aerosol inhalation of HRS protected the kidneys and increased survival among septic mice. HRS was confirmed to promote M2 macrophage polarization in lipopolysaccharide-stimulated RAW 264.7 cells. The TGF-β1 receptor inhibitor SB-431542 partly reversed the effects of HRS on renal function, fibrosis, tubular epithelial cell apoptosis and senescence in mice. Thus, HRS aerosol inhalation appears highly useful for renal protection and inflammation reduction in septic AKI.

## INTRODUCTION

During sepsis, the inflammatory process will become increasingly severe and ultimately lead to organ dysfunction if sepsis cannot be halted [1]. The kidney is the first and principle vital organ to be damaged by sepsis [2]. Acute kidney injury (AKI) is common in the early stages of sepsis, with an incidence of 55–73% [3, 4], and is independently associated with mortality [5]. Approximately 70% of septic AKI cases are fatal; thus, kidney protection is greatly important for the survival of sepsis patients [2]. Although many studies have explored the prevention and treatment of septic AKI, effective preventive treatments are still lacking [6–8].

An excessive inflammatory response and oxidative stress are considered to be the main mechanisms of septic AKI [9, 10]. Inflammation resolution is an important endogenous protective mechanism that can restore homeostasis and limit excessive tissue damage in response to injury [11, 12]. Any breakdown in the resolution of acute inflammation can lead to chronic renal inflammation, tissue destruction and renal fibrosis, ultimately causing renal failure [13]. Inflammation resolution is mainly characterized by neutrophil apoptosis, neutrophil elimination by macrophages, reduced secretion of pro-inflammatory cytokines and increased release of anti-inflammatory cytokines and pro-inflammatory resolution mediators [14]. Recently, promoting inflammation resolution was found to be critical for comprehensive sepsis therapy, suggesting a new therapeutic concept for preventing septic AKI [15]. Macrophage polarization from pro-inflammatory M1 to anti-inflammatory M2 macrophages has been identified as a key mechanism of inflammation resolution, and thus is an attractive target for septic AKI prevention [16].

Hydrogen is an effective anti-inflammatory antioxidant. In experimental animal studies and clinical trials, 1-2% hydrogen has been reported to protect the brain, heart, liver and intestines from inflammation and oxidative damage [17–21]. However, the clinical application of hydrogen has been limited due to safety issues. In recent years, the use of pressure to dissolve hydrogen in water has enabled the safe application of hydrogen in a hydrogen-rich solution (HRS) [22]. An *in vitro* study demonstrated that a 0.19-M HRS reduced A549 alveolar cell damage by inhibiting interleukin (IL)-6 and vascular endothelial growth factor cytokine production [23]. However, an effective and reliable route of drug administration has not yet been found for the HRS, and the molecular mechanisms behind its anti-inflammatory effects have not been elucidated.

In the current study, we tested the hypothesis that aerosol inhalation of a HRS would be a safe and effective method of delivering hydrogen, and would resolve renal inflammation during septic AKI by promoting macrophage polarization from pro-inflammatory M1 to anti-inflammatory M2 macrophages.

## RESULTS

### Aerosol inhalation of a HRS did not reduce the PaO_2_ level

To evaluate the safety of HRS inhalation, we administered an HRS to mice via aerosol inhalation for two hours, and observed their mental state, local respiratory responses and arterial blood gas levels. The mice were compliant with the inhalation procedure and were in a good mental state after two-hour aerosol inhalation of the HRS. No abnormal secretions were detected in the eyes or nasal cavity, and no obvious bleeding or foamy exudation was observed in the trachea or lungs. Arterial blood gas analysis (Table 1) revealed that the partial pressure of oxygen (PaO_2_) and oxygen saturation (SaO_2_) were slightly higher in mice administered the HRS than in those administered saline by aerosol inhalation, but the difference was not statistically significant (*P>0.05*). These results indicated that aerosol inhalation of a HRS is a feasible and safe method of delivering hydrogen without irritating the respiratory tract or reducing PaO_2_ levels. For subsequent experiments, we administered the HRS via aerosol inhalation for only one hour.

**Table 1:** Hydrogen Rich Solution (HRS) aerosol inhalation did not cause drop of PaO_2_.

**Index**	**Control**	**NS-inhalation**	**HRS-inhalation**	**F**	***P***
Weight (g)	24.4±1.2	24.8±1.3	25.0±1.1	0.1935	0.829
PaO_2_ (mmHg)	74.7±13.3	86.7±27.3	80.0±7.8	0.331	0.731
SaO_2_ (%)	89.7±6.7	91.3±5.5	92.7±2.1	0.257	0.781

*Note: n=3* per group, data were showed as *mean + SEM*, Significance was calculated using a *one-way ANOVA* analysis. Abbreviations: Control: blank control group, NS-inhalation: mice treated with NS ultrasonic aerosol inhalation, HRS-inhalation: mice treated with HRS ultrasonic aerosol inhalation; PaO_2_: arterial oxygen pressure, SaO_2_: arterial oxygen saturation.

### Aerosol inhalation of a HRS restored renal function and protected the kidneys from septic injury

**Figure 1:**
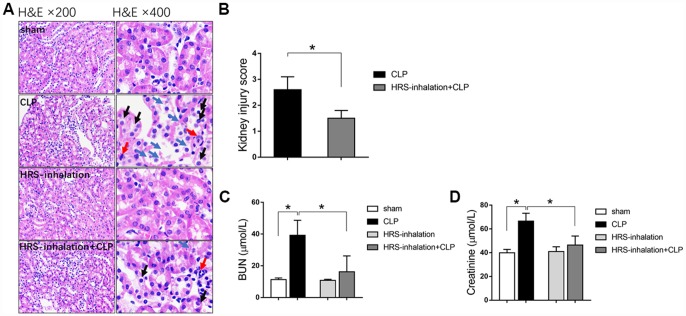
**Aerosol inhalation of an HRS restored renal function and protected the kidneys from septic injury**. (**A**) Hematoxylin and eosin staining; (**B**) Kidney injury scores; (**C**) BUN concentrations; (**D**) Serum creatinine concentrations. Blue arrows indicate tubular necrosis, red arrows indicate inflammatory cells, and black arrows indicate cell swelling and cytoplasm rarefaction. *n=6* per group. Data are shown as the *mean ± SEM*. In (**C**) and (**D**), significance was calculated by *one-way ANOVA* with *Tukey’s post hoc test*, *: P<0.05.

We generated a mouse model of septic AKI through a cecal ligation and puncture (CLP) operation. To study the effects of the HRS on septic AKI, we established four groups of mice: a sham operation group, a CLP group, a HRS inhalation group, and a HRS inhalation + CLP group. In our mouse model, AKI occurred in the early stage of sepsis. To evaluate the degree of kidney injury, we performed hematoxylin and eosin staining on renal pathological sections. The septic kidneys exhibited obvious pathological changes, including bleb formation, tubular necrosis, inflammatory cell infiltration, cell swelling, cytoplasm rarefaction, loss of the brush border, tubular luminal debris and obstruction (Figure 1A and 1B). The blood urea nitrogen (BUN) (Figure 1C) and serum creatinine (Figure 1D) concentrations were significantly elevated in the mice with septic AKI, reflecting their impaired renal function. Aerosol inhalation of the HRS for one hour prevented the changes in renal pathology, BUN and serum creatinine in mice that underwent the CLP (*P<0.05*), but did not alter these pathological and biochemical parameters in mice subjected to a sham operation. These results indicated that aerosol inhalation of a HRS protected the kidneys during the early stage of septic AKI.

### Aerosol inhalation of a HRS inhibited renal tubular epithelial cell apoptosis and senescence in mice subjected to sepsis

**Figure 2:**
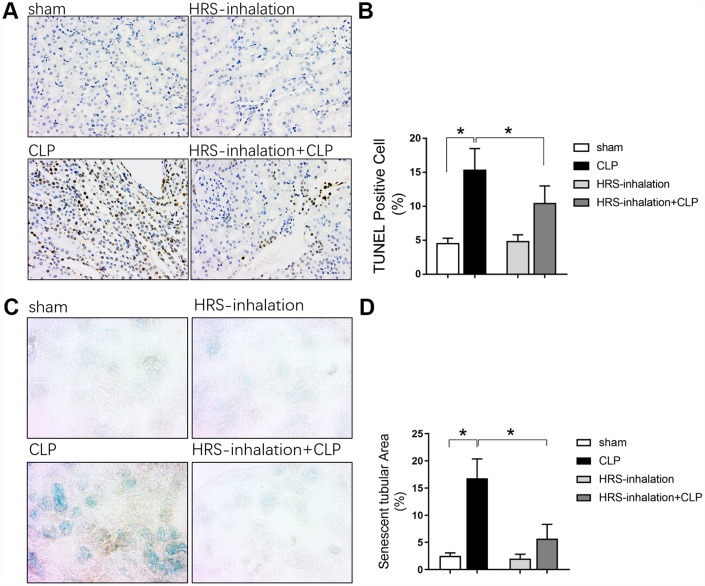
**Aerosol inhalation of an HRS inhibited renal tubular epithelial cell apoptosis and senescence in septic mice.** (**A**) TUNEL staining (×200); (**B**) Percentage of TUNEL-positive cells; (**C**) β-galactosidase staining (×400); (**D**) Percentage of senescent tubular area. *n=6* per group. Data are shown as the *mean ± SEM*. In (**B**) and (**D**), significance was calculated by *one-way ANOVA* with *Tukey’s post hoc test*, *: P<0.05.

Renal tubular epithelial cells are important for renal function. To explore the underlying mechanism of septic AKI, we performed terminal deoxynucleotidyl transferase dUTP nick end labeling (TUNEL) and β-galactosidase staining to detect renal tubular epithelial cell apoptosis and senescence, respectively, in the four groups of mice. Apoptosis and senescence increased rapidly in the early stage after sepsis attack, as evidenced by the significantly greater percentage of TUNEL-positive renal tubular epithelial cells (Figure 2A and 2B) and senescent tubular area (Figure 2C and 2D) in the CLP group than in the sham group (*P<0.05*). Aerosol inhalation of the HRS dramatically reduced the percentage of TUNEL-positive renal tubular epithelial cells (Figure 2A and 2B) and the senescent tubular area (Figure 2C and 2D) (*P<0.05* vs. CLP group). These results indicated that aerosol inhalation of the HRS may have protected the kidneys by attenuating renal tubular epithelial cell injury.

### Aerosol inhalation of an HRS attenuated sepsis-induced renal fibrosis

**Figure 3:**
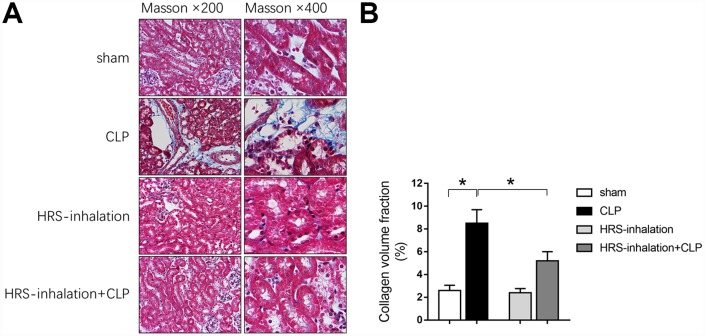
**Aerosol inhalation of an HRS attenuated sepsis-induced renal fibrosis.** (**A**) Masson’s Trichrome staining, where blue staining represents extracellular matrix deposition, suggesting fibrosis; (**B**) Collagen volume fraction ratio. *n=6* per group. Data are shown as the *mean ± SEM*. In (**B**), significance was calculated by *one-way ANOVA* with *Tukey’s post hoc test*, *: P<0.05.

We next performed Masson’s Trichrome staining to assess fibrosis formation in the kidneys of the four groups of mice. Kidney fibrosis increased significantly during early sepsis, as evidenced by the greater blue staining of extracellular matrix deposits in the CLP group than in the sham group (*P<0.05*) (Figure 3A and 3B). Aerosol inhalation of the HRS reduced the blue staining in the kidney cortex in mice subjected to CLP (*P<0.05* vs. CLP group) (Figure 3A and 3B), but did not alter the blue staining in the sham operation group. These results suggested that aerosol inhalation of the HRS may have protected the kidneys by retarding the progression of renal fibrosis.

### Macrophage infiltration contributed to septic AKI

**Figure 4:**
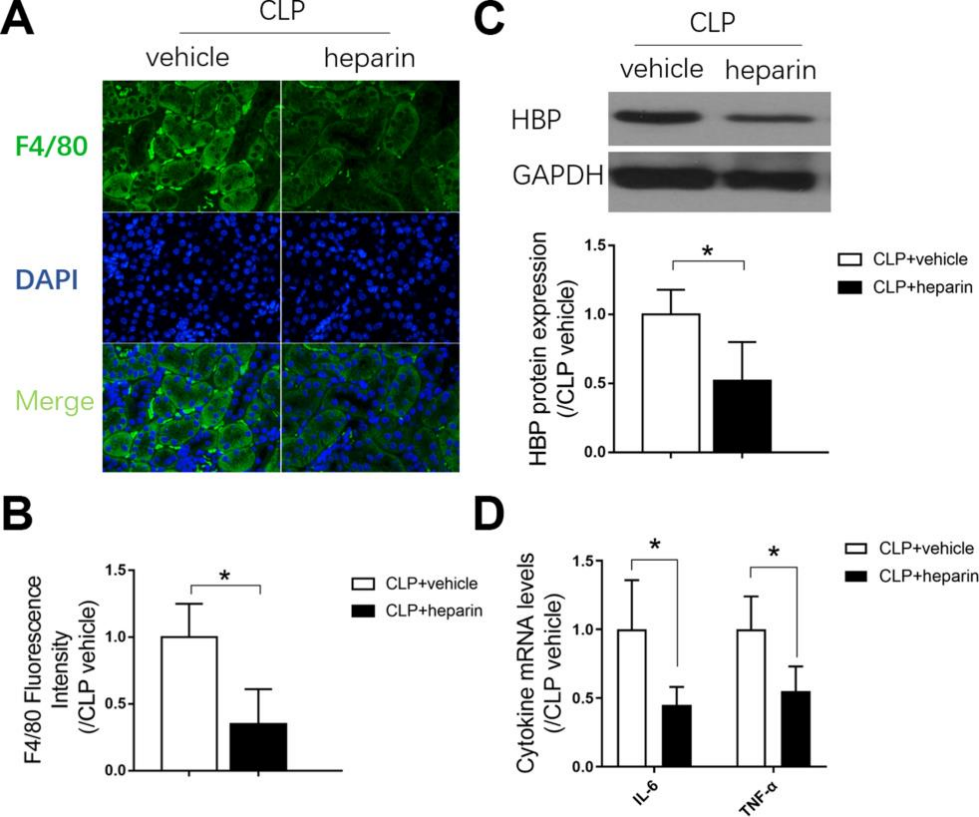
**The role of macrophage infiltration in septic AKI.** (**A**) F4/80 immunofluorescent staining (×400); (**B**) gray value for F4/80 immunofluorescent staining; (**C**) protein expression of heparin binding protein, and gray value for the Western blot band; (**D**) Renal tissue mRNA expression of *Il-6* and *Tnf-α*. *n=6* per group. Data are shown as the *mean ± SEM*. Significance was calculated by *one-way ANOVA* with *Tukey’s post hoc test*, *: P<0.05. Abbreviations: CLP + vehicle, the group in which mice underwent the cecal ligation and puncture operation; CLP + heparin, the group in which mice were treated with heparin before the cecal ligation and puncture operation.

Macrophages are important components of the immune defense system. We performed F4/80 cell staining to evaluate macrophage infiltration into the septic kidneys, and found that F4/80 positivity greatly increased after CLP (Figure 4A and 4B). To confirm the involvement of macrophages in septic AKI, we injected mice with a macrophage infiltration inhibitor (heparin) 12 hours before the CLP procedure. Heparin inhibited macrophage infiltration into the septic kidneys (Figure 4A and 4B) and repressed heparin binding protein expression in kidney tissues (Figure 4C). As macrophages are the main immune cells that produce inflammatory cytokines, we measured the mRNA expression of inflammatory cytokines (*Il-6* and tumor necrosis factor alpha [*Tnf-α*]) in the kidneys of mice treated with heparin or the vehicle before CLP. Heparin significantly reduced the expression of both cytokines (by nearly 50% compared with the CLP + vehicle group) (Figure 4D). These results indicated that macrophages infiltrated and produced inflammatory cytokines in septic kidneys, ultimately causing renal dysfunction.

### Aerosol inhalation of an HRS altered macrophage polarization in septic kidneys

**Figure 5:**
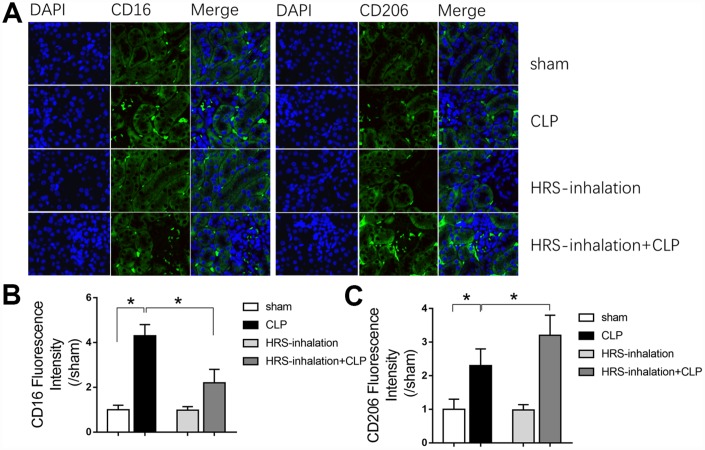
**Aerosol inhalation of an HRS altered macrophage polarization in septic kidneys.** (**A**) CD16 and CD206 immunofluorescent staining (×400); (**B**) gray value for CD16 immunofluorescent staining; (**C**) gray value for CD206 immunofluorescent staining. *n=6* per group. Data are shown as the *mean ± SEM.* In (**B**) and (**C**), significance was calculated by *one-way ANOVA* with *Tukey’s post hoc test*, *: P<0.05.

Having established the involvement of macrophages in kidney inflammation, we next assessed whether the renal protective effects of HRS inhalation were due to macrophage activity. We used CD16 and CD206 immunofluorescent staining to detect M1- and M2-type macrophage recruitment, respectively, in the kidneys of the four previously established groups of mice. As shown in Figure 5A, M1- and M2-type macrophages were both recruited to the septic renal cortex in the CLP group (*P<0.05* vs. sham group); however, aerosol inhalation of the HRS altered macrophage polarization by greatly reducing the proportion of M1-type macrophages and increasing the proportion of M2-type macrophages in the renal cortex (*P<0.05* vs. CLP group) (Figure 5A–5C). Aerosol inhalation of the HRS itself had no effect on macrophage polarization in the sham operation group. These results indicated that aerosol inhalation of the HRS may have reduced renal fibrosis by altering macrophage polarization and promoting M2-type macrophage recruitment.

### Aerosol inhalation of a HRS increased M2 macrophage-associated anti-inflammatory cytokine levels and reduced M1 macrophage-associated pro-inflammatory cytokine levels in septic kidneys

**Figure 6:**
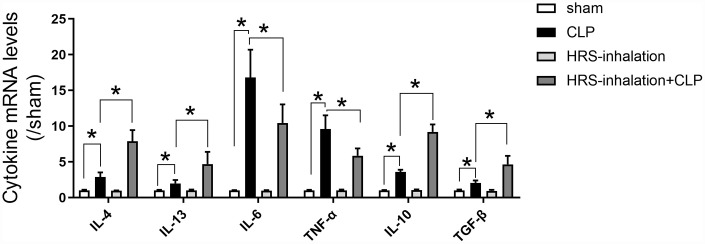
**Aerosol inhalation of an HRS increased M2 macrophage-associated anti-inflammatory cytokine levels and reduced M1 macrophage-associated pro-inflammatory cytokine levels in septic kidneys.** Renal tissue mRNA expression of *Il-4*, *Il-13*, *Il-6*, *Tnf-α*, *Il-10* and *Tgf-β*. *n=6* per group. Data are shown as the *mean ± SEM*. Significance was calculated by *one-way ANOVA* with *Tukey’s post hoc test*, *: P<0.05.

To explore the mechanism whereby aerosol inhalation of the HRS altered macrophage polarization in septic AKI, we assessed the mRNA levels of cytokines in renal tissues from the four groups of mice. The mRNA levels of *Il-4*, *Il-13*, *Il-6*, *Tnf-α*, *Il-10* and transforming growth factor beta (*Tgf-β*) in the kidneys were significantly greater in the CLP group than in the sham group (*P<0.05*) (Figure 6). *Il-4* and *Il-13* levels were greater in the HRS inhalation + CLP group than in the CLP group. Importantly, M1 macrophage-associated pro-inflammatory cytokine (*Il-6* and *Tnf-α*) mRNA levels were lower and M2 macrophage-associated anti-inflammatory cytokine (*Il-10* and *Tgf-β*) levels were higher in the HRS inhalation + CLP group than in the CLP group (*P<0.05*) (Figure 6). These results suggested that aerosol inhalation of the HRS elevated *Il-4* and *Il-13* expression and shifted macrophage polarization toward M2 macrophage activation, ultimately changing the cytokine profile.

### Aerosol inhalation of a HRS improved the survival rate of septic mice

**Figure 7:**
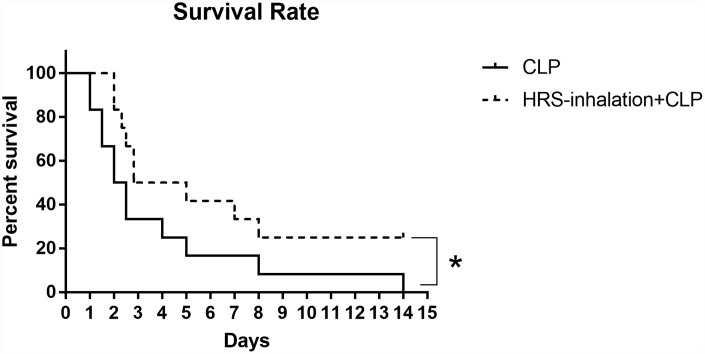
**Survival rate after HRS inhalation.** The survival rate was observed for 15 days after the CLP operation. *n=12*, *: P<0.05.

We then used Kaplan-Meier survival curves to estimate the survival rates of mice subjected to sepsis. The survival rate was higher in the HRS inhalation + CLP group (50%) than in the CLP group (33.3%) during the first 72 hours after the operation (Figure 7). All the mice in the CLP group died within 15 days, while 25% of the mice in the HRS inhalation + CLP group survived. Thus, the survival rate was significantly higher in the HRS inhalation + CLP group (*P<0.05*).

### Hydrogen-rich medium altered macrophage polarization in RAW 264.7 cells after induction

**Figure 8:**
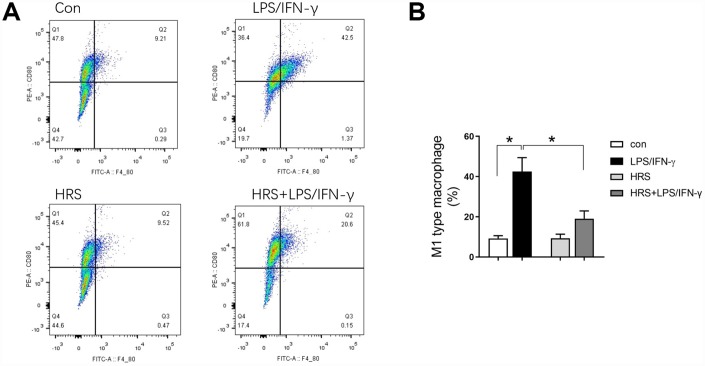
**Hydrogen-rich medium reduced M1-type macrophage polarization in RAW 264.7 cells after induction.** Flow cytometry of F4/80, CD80 double-positive macrophages (i.e., M1-type macrophages). *n=4* per group. Data are shown as the *mean ± SEM*. Significance was calculated by *one-way ANOVA*, *: P<0.05. Abbreviations: Con, control group; LPS/IFN-γ, RAW 264.7 cells treated with LPS and IFN-γ to induce polarization into M1-type macrophages; HRS, RAW 264.7 cells treated with hydrogen-rich medium only; HRS + LPS/IFN-γ, RAW 264.7 cells treated with hydrogen-rich medium after being treated with LPS and IFN-γ.

**Figure 9:**
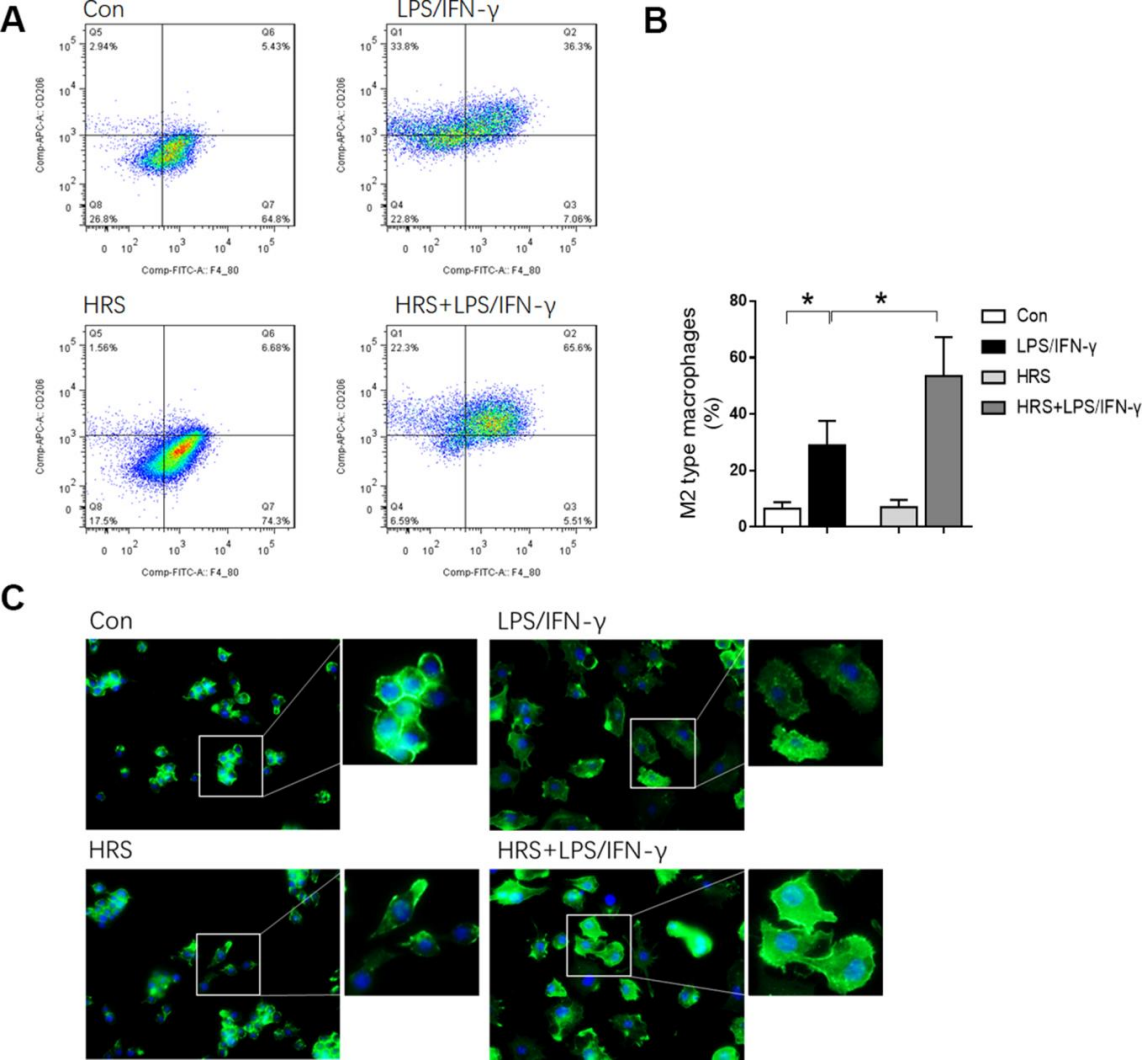
**Hydrogen-rich medium increased M2-type macrophage polarization in RAW 264.7 cells after induction, and increased the capture ability of macrophages.** (**A**) Flow cytometry of F4/80, CD206 double-positive macrophages (i.e., M2-type macrophages); (**B**) Fluorescent staining with ghostpen cyclopeptide on macrophages. *n=4* per group. Data are shown as the *mean ± SEM*. Significance was calculated by *one-way ANOVA*, *: P<0.05. Abbreviations: Con, control group; LPS/IFN-γ, RAW 264.7 cells treated with LPS and IFN-γ to induce polarization into M1-type macrophages; HRS, RAW 264.7 cells treated with hydrogen-rich medium only; HRS + LPS/IFN-γ, RAW 264.7 cells treated with hydrogen-rich medium after being treated with LPS and IFN-γ.

We then investigated the effects of hydrogen on macrophage polarization *in vitro* using RAW 264.7 cells. The cells were incubated in hydrogen-rich medium, and 1 μg/mL lipopolysaccharide (LPS) and 20 ng/mL interferon gamma (IFN-γ) were added to induce M1-type macrophage polarization. We then performed flow cytometry, using F4/80, CD80 as an M1 macrophage marker, and F4/80, CD206 as an M2 macrophage marker. A greater proportion of RAW 264.7 cells were polarized to M1- and M2-type macrophages in the LPS/IFN-γ treatment group than in the control group (*P<0.05*) (Figures 8A and 8B, 9A and 9B). However, the proportion of M1-type macrophages was significantly lower and the proportion of M2-type macrophages was significantly higher in the hydrogen-rich-medium-treated LPS/IFN-γ group than in the LPS/IFN-γ group (*P<0.05*). Moreover, fluorescent staining with ghostpen cyclopeptide revealed that the hydrogen-rich medium increased the capture ability of macrophages by enhancing the extensibility of their pseudopods (Figure 9C). These results were consistent with our *in vivo* findings, validating the notion that the HRS promoted macrophage polarization.

### Hydrogen-rich medium increased M2 macrophage-associated anti-inflammatory cytokine levels and reduced M1 macrophage-associated pro-inflammatory cytokine levels in RAW 264.7 cells after induction

**Figure 10:**
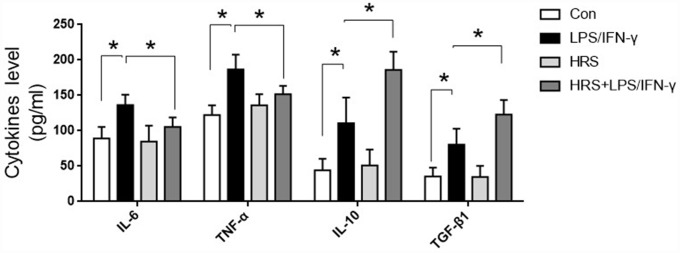
**Hydrogen-rich medium increased M2 macrophage-associated anti-inflammatory cytokine levels and reduced M1 macrophage-associated pro-inflammatory cytokine levels in RAW 264.7 cells after induction. Secretion of IL-6, TNF-α, IL-10 and TGF-β1 from macrophages. *n=8* per group.** Data are shown as the *mean ± SEM*. Significance was calculated by *one-way ANOVA*, *: P<0.05. Abbreviations: Con, control group; LPS/IFN-γ, RAW 264.7 cells treated with LPS and IFN-γ to induce polarization into M1-type macrophages; HRS, RAW 264.7 cells treated with hydrogen-rich medium only; HRS + LPS/IFN-γ, RAW 264.7 cells treated with hydrogen-rich medium after being treated with LPS and IFN-γ.

To investigate the effects of the hydrogen-rich medium on macrophage-associated cytokines, we used an enzyme-linked immunosorbent assay (ELISA) to detect cytokine levels in the culture supernatants of RAW 264.7 cells after induction. The levels of IL-6, TNF-α, IL-10 and TGF-β1 in the supernatant were significantly greater in the LPS/IFN-γ group than in the control group (*P<0.05*) (Figure 10). M1 macrophage-associated pro-inflammatory cytokine (IL-6 and TNF-α) levels were lower in the hydrogen-rich-medium-treated LPS/IFN-γ group than in the LPS/IFN-γ group, while M2 macrophage-associated anti-inflammatory cytokine (IL-10 and TGF-β1) levels were higher in the hydrogen-treated group (*P<0.05*). These results suggested that the hydrogen-rich medium altered cytokine levels and promoted the polarization of macrophages to the M2 type.

### TGF-β1 receptor blockage partly reversed the renal protective effects of HRS inhalation

**Figure 11:**
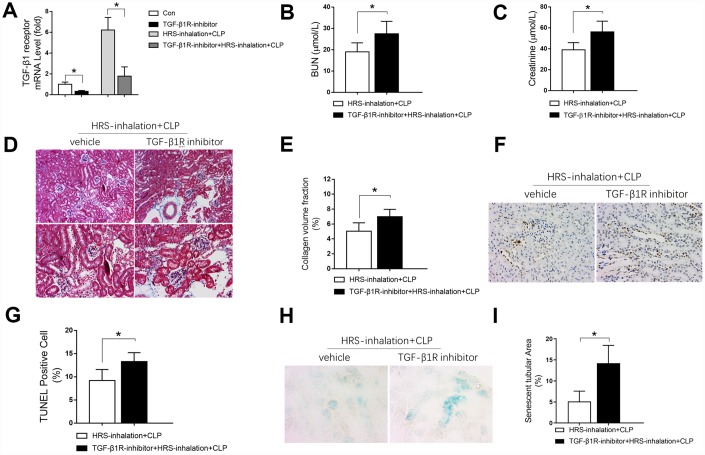
**Blockage of the TGF-β1 receptor partly reversed the renal protective effects of the HRS against septic AKI.** (**A**) *Tgf-β1 receptor* mRNA expression; (**B**) BUN concentrations; (**C**) Serum creatinine concentrations; (**D**) Masson’s Trichrome staining, where blue staining represents extracellular matrix deposition, suggesting fibrosis; (**E**) Collagen volume fraction ratio; (**F**) TUNEL staining (×200); (**G**) Percentage of TUNEL-positive cells; (**H**) β-galactosidase staining (×400); (**I**) Percentage of senescent tubular area. *n=6* per group. Data are shown as the *mean ± SEM*. Significance was calculated by *one-way ANOVA* with *Tukey’s post hoc test*, *: P<0.05. Abbreviations: Con, the group in which mice were treated with the vehicle; TGF-β1R inhibitor, the group in which mice were treated with the TGF-β1 receptor inhibitor only; HRS inhalation + CLP, the group in which mice were treated with HRS aerosol inhalation after the cecal ligation and puncture operation; TGF-β1R inhibitor + HRS inhalation + CLP, the group in which mice were pretreated with the TGF-β1 receptor inhibitor before HRS aerosol inhalation and the cecal ligation and puncture operation.

We then investigated whether cytokine receptors contributed to the effects of HRS inhalation in septic AKI. As shown in Figure 11A, HRS inhalation increased renal *Tgf-β1 receptor* expression during CLP. Accordingly, pretreatment with the TGF-β1 receptor inhibitor SB-431542 effectively prevented the increase in *Tgf-β1 receptor* expression upon HRS inhalation in CLP mice (Figure 11A). Moreover, inhibiting the TGF-β1 receptor increased the BUN levels (Figure 11B), serum creatinine levels (Figure 11C), renal tissue collagen volume fraction (Figure 11D and 11E), percentage of TUNEL-positive renal tubular epithelial cells (Figure 11F and 11G) and senescent tubular area (Figure 11H and 11I) in the HRS + CLP mice. Thus, inhibiting the TGF-β1 receptor partly reversed the effects of the HRS on renal function, fibrosis, tubular epithelial cell apoptosis and senescence in septic mice. These results indicated that HRS inhalation exerted renal protective effects mainly by enhancing anti-inflammatory cytokine expression.

## DISCUSSION

Septic AKI aggravates the systemic inflammatory response and leads to multiple organ failure [24]. In the current study, we evaluated the safety and effectiveness of aerosol inhalation of an HRS in septic AKI. A CLP operation was performed in mice to mimic clinical sepsis, and AKI was found to occur in the early stage of sepsis. We administered an HRS via aerosol inhalation *in vivo*, and found that it protected renal function, prevented renal fibrosis and inhibited renal tubular epithelial cell apoptosis in mice subjected to CLP. The HRS exerted these effects by facilitating macrophage polarization from the M1 to the M2 phenotype, thus resolving renal inflammation.

The HRS, a novel hydrogen-saturated physiological saline solution, was previously reported to inhibit CLP-induced inflammatory responses, oxidative stress and apoptosis when injected intraperitoneally [25]. Shingu et al. also found that intravenous administration of an HRS reduced renal ischemia/reperfusion injury in rats [26]. Shigeta et al. reported that luminal injection of an HRS attenuated oxidative stress and thus ameliorated ischemia/reperfusion injury in a small bowel transplantation model in rats [27]. The three methods of HRS administration used in the above studies (intraperitoneal, intravenous and luminal) all carry the risk of aeroembolism, since hydrogen is an insoluble gas. Thus, we designed the HRS for delivery via aerosol inhalation so that it could be absorbed through the pulmonary microcirculation. Aerosol inhalation has been proven to be a safe and fast-acting therapeutic method in critical illness [28]. In the present study, aerosol inhalation of the HRS protected the kidneys from septic AKI by preserving renal function and preventing renal pathology, fibrosis and tubular epithelial cell apoptosis. These results suggested that aerosol inhalation of a HRS is a safe and effective method of treating septic AKI.

Inflammation resolution involves the generation of pro-resolving mediators and the relief of inflammation [29, 30]. M2-polarized macrophages are critical components of inflammation resolution [31, 32]. We found that aerosol inhalation of an HRS resolved inflammation by increasing M2 macrophage polarization. The process of M2 macrophage polarization depends on cytokines. Activation of the IL-4/IL-13 signaling pathway was reported to promote renal M2 macrophage polarization, and the percentage of renal M2-activated macrophages after diphtheria toxin injection was found to be greatly reduced in IL-4/IL-13-knockout mice [33]. In our study, the mRNA levels of *Il-4* and *Il-13* in renal tissues increased following aerosol inhalation of the HRS, indicating that the HRS may have activated M2 macrophage polarization by stimulating the IL-4/IL-13 signaling pathway. Macrophage polarization is beneficial for renal injury repair because M2 macrophages can both secrete anti-inflammatory molecules and produce growth factors that promote tissue repair and regeneration [34]. We found that the mRNA levels of the anti-inflammatory cytokine *Il-13* and the growth factor *Tgf-β* in the kidneys increased after aerosol inhalation of the HRS, suggesting that the HRS promoted M2 macrophage polarization and cytokine secretion, ultimately protecting the kidneys.

Of note, although aerosol inhalation of the HRS was effective in the present study, the inhaled concentration of the HRS was not detected. Since the aerosol HRS was inhaled into the lungs, it would have been necessary to detect the H_2_ concentration in the lungs via the trachea. A sensor to detect H_2_ in the lungs is not yet available in the biological instrument market, although we may develop this tool in the future. Also, H_2_ will be released from the blood quickly when blood is collected by normal approaches, because atmospheric pressure is below the pressure at which hydrogen dissolves (about six atmospheric pressure units). Thus, a real-time monitoring instrument to determine the blood H_2_ concentration also needs to be developed in the future. Additionally, although aerosol inhalation of the HRS prevented renal tubular epithelial cell apoptosis by suppressing inflammation, further *in vitro* experiments are required to determine whether the HRS also directly protected renal tubular epithelial cells from septic injury.

**Figure 12:**
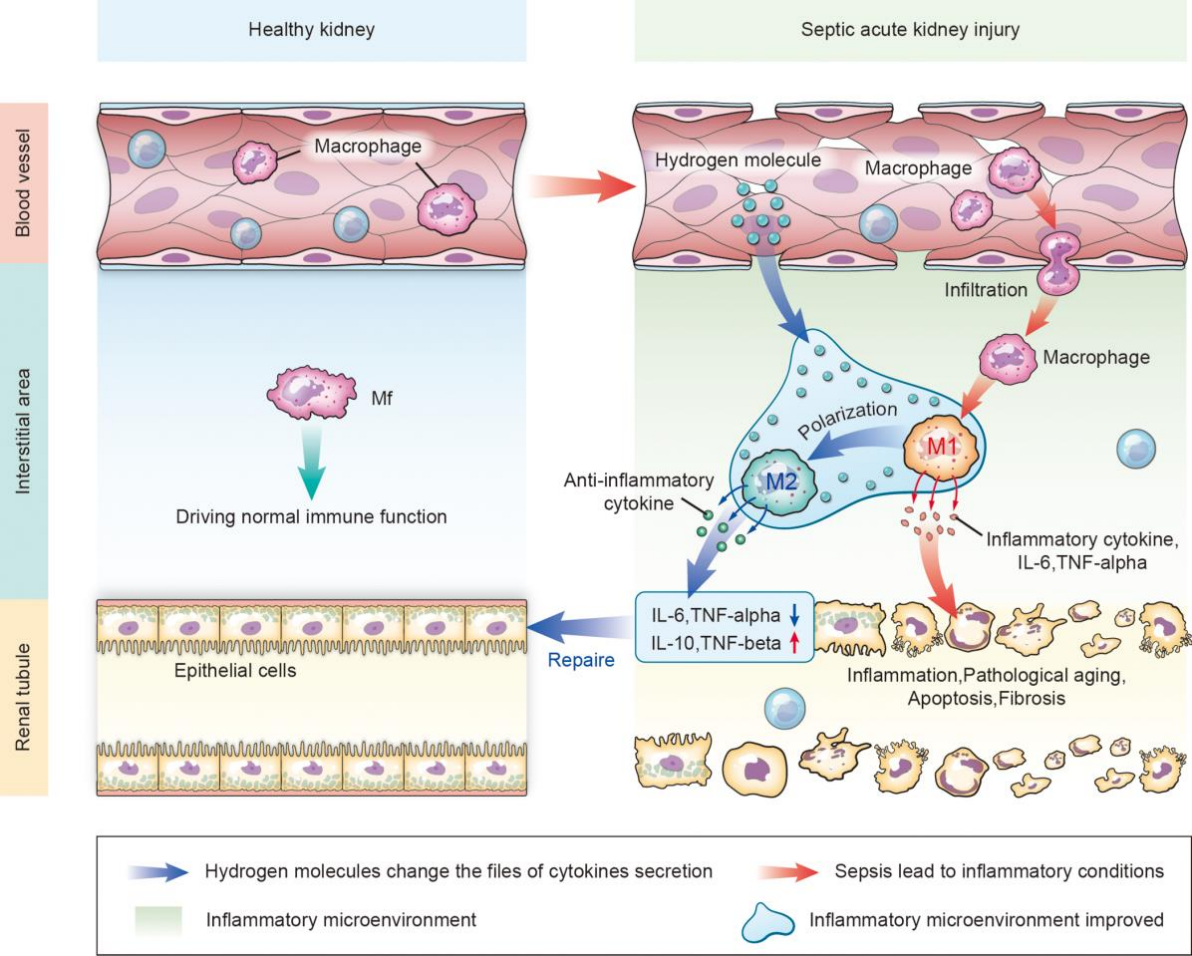
**Schematic diagram of how aerosol inhalation of the HRS attenuated septic renal injury by promoting macrophage M1/M2 polarization.** Normally, macrophages (Mf) perform immune functions in the interstitium of the kidney. In the case of kidney injury, the permeability of the vascular endothelial cells increases. Intravascular macrophages infiltrate the interstitial spaces and form M1-type macrophages that secrete inflammatory cytokines, thus destroying renal tubular epithelial cells. After HRS treatment, hydrogen molecules can infiltrate the tissues through the blood vessels, change the inflammatory factors that are secreted in the inflammatory environment, and promote the polarization from M1-type to M2-type macrophages. M2 macrophages secrete anti-inflammatory factors to repair the damaged renal tubules.

In summary, aerosol inhalation of an HRS significantly protected kidney function, prevented renal pathology and inhibited fibrosis during septic AKI. The therapeutic efficacy of the HRS appeared to result from its resolution of renal inflammation, particularly through its alteration of the macrophage cytokine profile and its promotion of macrophage polarization to the M2 phenotype (Figure 12).

## MATERIALS AND METHODS

### Animals

This study was approved by the Sun Yat-sen University Animal Experimentation Committee. Animal care was performed in accordance with the National Institutes of Health criteria for the care and use of experimental animals. Eight-week-old male specific-pathogen-free C57BL/6 mice were purchased from Guangdong Medical Experimental Animal Center (Guangzhou, China). The mice were fasted for 12 hours before the experiments, but were allowed access to water.

### Cell culture

RAW 264.7 cells (mouse mononuclear macrophage leukemia cells) were purchased from the American Type Culture Collection and cultured in Dulbecco’s modified Eagle’s medium supplemented with 10% fetal bovine serum and 1% penicillin/streptomycin. Cells were grown at 37 °C in an atmosphere of 5% CO_2_ in air.

### HRS preparation

Hydrogen produced by a hydrogen generator (ZK-300, Beijing Zhongke Hui Heng Technology Development Company, Beijing, China) was dissolved in physiological saline or medium under 4 atmospheres for six hours (i.e., to a hyper-saturated state) in our laboratory. The hydrogen content of the resulting HRS was detected with a dissolved hydrogen detector (ENH-1000, Dalian Xin Qiang Trade Limited Company, Dalian, China) to ensure that the concentration was above 0.6 mmol/L. The HRS was freshly prepared before each experiment.

### Ultrasonic aerosol inhalation

A medical ultrasonic nebulizer (HXHL-1, Guangzhou Weili Medical Devices Company, Guangzhou, China) was connected with a ventilator and a homemade box. The fresh airflow released by the ventilator slightly pushed a one-way valve and was detected by a photoelectric sensor, and then ultrasonic aerosol inhalation started.

### Establishment of the mouse CLP model

The mouse CLP model was established as described previously [35]. Briefly, mice were anesthetized with a mixture of chlorpromazine and ketamine (1:5) and were fixed on an operating table in the supine position. After the skin was prepared and the operative area was disinfected, a ventral midline incision (1.5 cm) was made and the mesentery and cecum were freed with tweezers. The cecum was ligated with a No. 4 sterile silk ligature below the ileocecal valve, punctured through with a 20-gauge needle and restored to the abdomen after some excrement was squeezed out. The abdominal incision was then sutured layer by layer. The mice were fluid-resuscitated through a dorsal subcutaneous injection of 1 mL of physiological saline, and were allowed to eat and drink freely after the operation.

### Experimental design

Part 1: The mice were randomly divided into three groups: (1) the blank control group, (2) the saline inhalation group and (3) the HRS inhalation group. The mice in the HRS inhalation group inhaled the HRS for two hours, while the mice in the saline inhalation group inhaled an equivalent saline solution for two hours. At the end of the inhalation period, the mice were observed for their inhalation compliance and mental state, along with signs of suffocation or vomiting. The mental state was categorized into one of three levels: Good, the mice moved briskly, consumed water and food normally and had glossy hair; Average, the mice were less active, consumed less water and food and had matted hair; Energy loss, the mice were inert, did not drink or eat and had dull hair. During inhalation, we also assessed compliance as follows: Tolerant, the mice behaved normally, did not struggle and breathed smoothly; Average, the mice were active, struggled and increased their respiratory rate; Intolerant, the mice exhibited irritability and tachypnea. Then, the mice were anesthetized, and retro-orbital blood was collected for the detection of arterial blood gas with a blood analyzer (i-STAT 1 Analyzer, American Abbott). The local respiratory reactions of the mice after sacrifice were observed.

Part 2: The mice were randomly divided into four groups: (1) the sham-operated group (sham group), (2) the sepsis group (CLP group), (3) the HRS inhalation group and (4) the HRS inhalation + CLP group. The mice in the CLP group were subjected to the CLP procedure described above, while the mice in the sham group were subjected to the same operation without the cecal ligation and puncture. The mice in the HRS inhalation group and the HRS inhalation + CLP group inhaled the HRS (10 mL/kg, 0.6 mmol/L) for one hour after model establishment, while the mice in the sham group and the CLP group inhaled an equivalent saline solution. Eighteen hours after the CLP operation, the mice were anesthetized and retro-orbital blood was collected. The blood was left for one hour at room temperature, and then was centrifuged at 3000 rpm for 10 min. The serum was collected and frozen at -80 °C for subsequent detection. The abdomen was then opened along the ventral midline, and the aorta was separated and exposed for complete bloodletting. The bilateral kidneys were harvested and frozen at −80 °C for subsequent detection.

Part 3: To evaluate the involvement of macrophages in septic AKI, we injected mice with heparin (intraperitoneal, 0.4 U/g, Formumax, USA) or vehicle 12 hours prior to CLP. Eighteen hours after the CLP operation, the mice were anesthetized, and their kidneys were harvested and frozen at -80 °C for subsequent detection.

Part 4: Mice were subjected to CLP with or without HRS inhalation, and were observed every 24 hours for 15 days for survival analysis. Kaplan-Meier survival curves were used to estimate the survival rates of the two groups.

Part 5: RAW 264.7 cells in logarithmic growth phase were placed in six-well plates and divided into four groups: the control group, the induced M1-type macrophage group (LPS/IFN-γ group), the hydrogen-rich medium group (HRS group) and the induced M1-type macrophage and hydrogen-rich medium group (HRS + LPS/IFN-γ group). For the LPS/IFN-γ group, 1 μg/mL LPS and 20 ng/mL IFN-γ were added to the cells for 24 hours to induce their development into M1-type macrophages. The medium was replaced with hydrogen-rich medium before the same concentrations of LPS and IFN-γ were added to the cells of the HRS + LPS/IFN-γ group. Twenty-four hours after the treatment, the cells and supernatants were collected for flow cytometry and ELISA, respectively.

Part 6: The TGF-β1 receptor inhibitor SB-431542 (MedChem Express, Monmouth Junction, NJ, USA) was used to assess the mechanism underlying the effects of HRS inhalation. SB-431542 (0.5 mg/mL) or its vehicle (10% ethanol) was injected intraperitoneally (4.2 mg/kg) into the mice daily for seven consecutive days before the CLP operation. The mice were then subjected to the CLP operation and subsequent HRS inhalation. Eighteen hours after the CLP operation, the mice were anesthetized and their blood and kidneys were collected for subsequent detection.

### Pathology

Unilateral kidney tissues were fixed in 4% paraformaldehyde for 24 hours, embedded in paraffin, cut into sections and stained with hematoxylin and eosin. Histopathological changes in the kidney tissues were observed under an optical microscope. According to the kidney injury scoring standard [36], the kidney injury was scored as follows: 0=normal kidney tissue; 1=impaired kidney tubule area <25%; 2=impaired kidney tubule area 25–50%; 3=impaired kidney tubule area 50–75%; 4=impaired kidney tubule area >75%.

### Biochemical detection

Serum creatinine and BUN levels were analyzed with an automatic biochemical analyzer (Chemray 240, Lei Du Life Scientific and Technical Corporation of Shenzhen) according to the instructions for the instrument.

### Masson’s trichrome staining

Paraffin-embedded kidney tissues were cut into 5-μm-thick slices. The slices were stained with Masson's trichrome procedure as described previously [37].

### TUNEL detection

Dewaxed and hydrated paraffin-embedded kidney tissue sections were washed with phosphate-buffered saline (PBS) three times for 5 min each, and were then incubated with 20% normal bovine serum at room temperature for 30 min. The sections were incubated with the TUNEL reaction mixture at 37 °C for 90 min, washed with PBS three times for 5 min each, and then blocked with a 3% H_2_O_2_ methanol solution at room temperature for 10 min. Next, the sections were incubated at 37 °C for 90 min, incubated with a peroxidase conversion agent at 37 °C for 30 min, and stained with diaminobenzidine/H_2_O_2_. The cells with brown granules in their nuclei were considered to be TUNEL-positive cells. The TUNEL-positive cells and total cells in five high-power fields from the kidney sections of different mice were counted. The apoptotic index (%) was calculated as: TUNEL-positive cells/total cells × 100%.

### Immunofluorescence assay

Paraffin-embedded kidney tissues were cut into 5-μm-thick slices. The slices were dewaxed, hydrated, washed with PBS twice for 5 min each, and then incubated with a 3% H_2_O_2_ methanol solution at room temperature for 10 min after repair. The slices were incubated with an F4/80 monoclonal antibody (1:1000, ab240946, Abcam, USA), a CD16 monoclonal antibody (1:1000, ab25235, Abcam, USA), a CD206 monoclonal antibody (1:1000, No.141707, BD Pharmingen, USA) or PBS (the negative control) overnight at 4 °C. Then, the slices were incubated with fluorescein isothiocyanate (FITC)-labeled rat anti-goat IgG (1:1000) at 37 °C in the dark for one hour. Subsequently, the slices were sealed with glycerol and visualized under a fluorescence microscope. The FITC-labeled F4/80, CD16 and CD206 indirectly emitted blue-green light.

### β-galactosidase assay

The expression of β-galactosidase (SA-β-gal) in renal tissues was analyzed with a β-galactosidase staining kit (Beyotime, Shanghai, China) in accordance with the manufacturer’s protocols.

### Quantitative real-time PCR assay

Reverse transcription PCR and quantitative real-time PCR were carried out as previously described [38] to detect cytokine mRNA levels. The primer sequences were as follows: *Il-4* forward: 5′-GAATGTACCA GGAGCCATATC-3′, reverse: 5′-CTCAGTACTACG AGTAATCCA-3′; *Il-13* forward: 5′-TGAGGAGCTGA GCAACATCA-3′, reverse: 5′-ATTTTGGTATCGG GGAGGCT-3′; *Tnf-α* forward: 5′-GCCAGCCGAT GGGTTGTA-3′, reverse: 5′-GGCAGCCTTGTC CCTTGA-3′; *Tgf-β* forward: 5′-TGCGTCTGCTG AGGCTCAA-3′, reverse: 5′-TTGCTGAGGTATCG CCAGGA-3′; *Il-6* forward: 5'-CCACTTCACAAGT CGGAGGCTTA-3', reverse: 5'-GCAAGTGCATC ATCGTTGTTCATAC-3'; *Il-10* forward: 5'-TGGACAACATACTGCTAACCGA-3', reverse: 5'-ACCCAGGGAATTCAAATGC-3'; *Tgf-β1 receptor* forward: 5'-TGCGGTTATGGCAGATATAGACC-3', reverse: 5'-TAGCTGAAATTGACCTAATTCCTCG-3'; *Gapdh* forward: 5′-AACTTTGGCATTGTGGAAGG-3′, reverse: 5′-GGATGCAGGGATGTTCT-3′.

### Flow cytology

Twenty-four hours after treatment, the RAW 264.7 cells were washed twice with PBS, resuspended with a micropipette and centrifuged at 1500 rpm at 4 °C for 5 min. The supernatant was removed, and the cells were then incubated with (i) anti-mouse F4/80-FITC antibody and anti-mouse CD80-PE antibody or (ii) anti-mouse F4/80-FITC antibody and anti-mouse CD206-PE antibody at room temperature for 15 min in the dark. The cells were centrifuged again and transferred into a flow tube for flow cytometry analysis.

### ELISA

The levels of IL-6, TNF-α, IL-10 and TGF-β1 secreted into the supernatants of RAW 264.7 cells 24 hours after treatment were assessed with ELISA kits (KeyGEN BioTECH, Jiangsu, China). The IL-6, TNF-α, IL-10 and TGF-β1 levels were quantified according to the manufacturer’s protocols.

### Western blotting

Kidney tissues (100 mg) were homogenized, and a bicinchoninic acid protein assay was used to quantify the protein levels. A heparin binding protein primary antibody (1:1000, Abcam, USA) was used for Western blotting, which was performed as described in our previous study [39].

### Statistical analysis

Data were analyzed with SPSS 19.0 and GraphPad Prism 5.0 statistical software. Data are presented as the mean ± standard error of the mean (SEM), and the means of different groups were compared by one-way analysis of variance (ANOVA). Statistical significance was set at *P<0.05*.
